# Food Insecurity is Increasing and is More Common Among Persons with Chronic Liver Disease

**DOI:** 10.21203/rs.3.rs-4509890/v1

**Published:** 2024-06-17

**Authors:** Cindy W. Leung, Elliot B. Tapper

**Affiliations:** Harvard University; University of Michigan

**Keywords:** NAFLD, cirrhosis, obesity, food insecurity

## Abstract

**Background:**

Effective interventions for metabolic liver disease include optimized nutritional intake. It is increasingly clear, however, that many patients with metabolic liver disease lack the resources to execute nutritional advice. Data on the trends of food insecurity are needed to prioritize public health strategies to address the burden of liver disease.

**Methods:**

Cross-sectional analysis of six waves of data from the 2007–2018, 24,847 subjects aged ≥20 years from the 2017–2018 National Health and Nutrition Examination Survey. Food security was measured using the US Department of Agriculture’s Core Food Security Module. Liver disease was defined as elevated liver enzymes and a risk factor: elevated BMI, diabetes, and/or excess alcohol consumption. Models were adjusted using age, sex, race/ethnicity, education, poverty-income ratio, smoking, physical activity, alcohol intake, sugary beverage intake, Healthy Eating Inex-2015 score. Advanced liver disease was estimated using FIB-4 >2.67.

**Results:**

The overall prevalence of liver disease was 24.6%, ranging from 21.1% (2017–2018) to 28.3% (2015–2016) (*P-trend=0.85*). 3.4% of participants had possible advanced liver disease, ranging from 1.9% (2007–2008) to 4.2% (2015–2016)(*P-trend=0.07*). Among those with liver disease, the prevalence of food insecurity was 13.6% in 2007–2008, which rose steadily to 21.6% in 2015–2016, before declining to 18.0% in 2017–2018 (*P-trend=0.0004*). Food insecurity rose more sharply for adults aged <50 years (2007–2008: 17.6%, 2015–2016: 28.0%, *P-trend=0.004*) compared to adults aged ≥50 years (2007–2008: 9.5%, 2015–2016: 16.5%, *P-trend*<*0.0001*). Food insecurity was more common among women, those with high BMI, and those with diabetes

**Conclusion:**

Food insecurity is increasingly common among those with liver disease.

## Introduction

Chronic liver disease (CLD) is a major threat to our public health.^1^ Roughly 60,000 people die from CLD annually.^2^ CLD accounts for more than 1 million outpatient visits and CLD-related hospitalizations are increasing annually, overtaking hospitalization-rates for heart failure and lung disease.^3,4^ Annual CLD healthcare costs exceed $29.9 billion.^5^ Increasingly, the most common etiology of CLD is metabolic-dysfunction associated steatotic liver disease (MASLD) which, in turn, most closely associates with obesity, insulin resistance, and, above all, poor dietary intake.^6–8^ Even among persons with excess alcohol intake and alcohol related liver disease (ALD), diet and obesity influence the risk of liver disease – the so-called ‘MetALD’. Effective interventions for metabolic liver disease include optimized nutritional intake. It is increasingly clear, however, that many patients with metabolic liver disease lack the resources to execute nutritional advice.^9^

A healthy diet presupposes accessibility and affordability of foods consistent with evidence-based diet patterns. Unfortunately, food insecurity is common, impacting 10.2% of households.^10–12^ Food insecurity, a condition of limited access to healthy food, has been associated with increased risks of obesity, diabetes, cardiovascular disease and indeed with metabolic liver disease.^12,13, 13^ Our previous study showed that food insecurity is associated with an increased risk of cirrhosis for persons aged > 50 years in a nationally representative sample.^9^ Kardashian has shown that food insecurity increased mortality for those with chronic liver disease.^14^ Food insecurity is an urgent problem.

Given that food insecurity is associated with metabolic liver disease, data regarding trends in food insecurity are needed. Such data would inform policy and justify funding for interventions which can both alleviate food insecurity and prevent progressive liver disease. We therefore used data from the 2007–2018 National Health and Nutrition Examination Survey (NHANES) to identify trends in food insecurity among persons with and without CLD over a two-year period.

## Methods

### Study population

We performed a serial cross-sectional analysis of six waves of data from the 2007–2018 NHANES. The NHANES is a nationally representative cross-sectional study conducted by the National Center for Health Statistics (NCHS) at the Centers for Disease Control and Prevention. NHANES enrolls participants using a stratified multistage probability with an oversampling design of certain age and racial/ethnic groups to allow for weighted analyses of the civilian non-institutionalized US population.^15^ All participants are interviewed for demographic, socioeconomic, dietary, and health information, and physical examinations and laboratory tests are conducted on most study participants. In the present study, the analytic sample included 24,847 adults ≥ 20 years, not pregnant at the time of the survey, and with complete data on food insecurity. We also excluded participants with chronic viral hepatitis based on positive hepatitis C virus (HCV) RNA or hepatitis B surface antigen (HBsAg). NHANES data are de-identified and publicly available; thus, no further IRB review was deemed necessary.

### Liver disease

We defined the presence of probably liver disease as having elevated liver enzymes and the presence of additional risk factors. Elevated liver enzymes were defined as high ALT or high AST (men ≥ 30 U/L and women ≥ 20 U/L). Additional risk factors included having a body mass index (BMI) ≥ 30 kg/m^2^, self-reported prior diagnosis of diabetes, or heavy alcohol consumption (current intake ≥ 7 drinks/week for women, ≥ 14 drinks/week for men). BMI was calculated from measured height and weight in the Mobile Examination Center (MEC). As secondary definitions, we also used advanced fibrosis on the basis of fibrosis-4 (FIB-4) index > 2.67. This was calculated as age (year) × AST (U/L)/(platelet count [109/L] × square root(ALT U/L).^14^

### Food Insecurity

Household food security over the past year was defined using the US Department of Agriculture’s (USDA) Core Food Security Module. This module includes 18 questions about food security addressing anxiety over the food supply, ability to eat a balanced meal, and behavioral manifestations of food rationing at the household, adult, and child levels. Individuals with > 3 affirmative responses are defined as food insecure; individuals with 0–2 affirmative responses are defined as food secure.

### Covariates

Sociodemographic characteristics of interest included age (20–29, 30–39, 40–49, 50–59, 60–69, 70–79, ≥ 80 years), sex (male, female), race and ethnicity (Non-Hispanic White, Non-Hispanic Black, Hispanic, Other), education level (high school degree or fewer years, any college), poverty income ratio (≤ 200% of the federal poverty level (FPL), > 200% FPL), and participation in the Supplemental Nutrition Assistance Program (SNAP) (yes, no). Health characteristics included BMI and elevated glycohemoglobin (< 6.5%, ≥ 6.5%). Heavy alcohol consumption, although used to help define the presence of liver disease, was not considered as a further covariate due to small sample sizes.

### Statistical analysis

NHANES MEC sampling weights were recalculated and used in all analyses to account for differential selection probabilities, nonresponse, and to make nationally representative estimates over the 12-year period. First, we estimated proportions of adults with food insecurity, liver disease, and demographic and health covariates stratified by survey wave, Chi-squared tests were used to examine differences in demographic and health covariates across survey waves. We calculated frequencies in food insecurity among adults with liver disease across survey waves in the overall population and stratified by sociodemographic and health covariates. We then estimated trends in food insecurity among adults with and without liver disease across survey waves, adjusted for covariates. In all analyses, we tested the significance of time trends by including survey wave as an ordinal variable in multivariate regression models. Finally, we fit a multivariable logistic regression model for the outcome of food insecurity including all sociodemographic and health covariates among adults with liver disease. All statistical analyses were performed using SAS, version 9.4.

## Results

### Participant characteristics

Across all waves, we included 24,847 adults > 20 years of age. The prevalence of food insecurity ranged from 11.5% (2007–2008) to 18.1% (2015–2016) (*P-trend*<*0.0001*) (Table 1). The overall prevalence of liver disease was 24.6%, ranging from 21.1% (2017–2018) to 28.3% (2015–2016) (*P-trend = 0.85*). In addition, 3.4% of participants had possible advanced liver disease, ranging from 1.9% (2007–2008) to 4.2% (2015–2016) (*P-trend = 0.07*). Across the twelve survey years, there were similar distributions of sex, race and ethnicity, marital status, and income to poverty ratio. Some significant differences in age category, educational attainment, BMI category, elevated glycohemoglobin, and SNAP participation was detected.

### Trends in food insecurity among adults with liver disease

In Table 2, we highlight the overall trends in food insecurity among adults with liver disease and stratified by demographic and health subgroups. Among those with liver disease, the prevalence of food insecurity was 13.6% in 2007–2008, which rose steadily to 21.6% in 2015–2016, before declining to 18.0% in 2017–2018 (*P-trend = 0.0004*). Table 2 also shows heterogeneity in these trends. Food insecurity rose more sharply for adults aged < 50 years (2007–2008: 17.6%, 2015–2016: 28.0%, *P-trend = 0.004*) compared to adults aged ≥ 50 years (2007–2008: 9.5%, 2015–2016: 16.5%, *P-trend*<*0.0001*). Similarly, food insecurity increased by 6.5% in women (*P-trend*<*0.0001*), compared to 2.6% in men (P-trend = 0.0007), and by 8.2% in Hispanic adults (*P-trend = 0.005*) compared to non-Hispanic White adults (*P-trend = 0.0002*). Food insecurity also increased significantly among those with incomes ≤ 200% FPL, from 25.2% in 2007–2008 to 38.5% in 2017–2018 (*P-trend*<*0.0001*).

**Figure 1:**
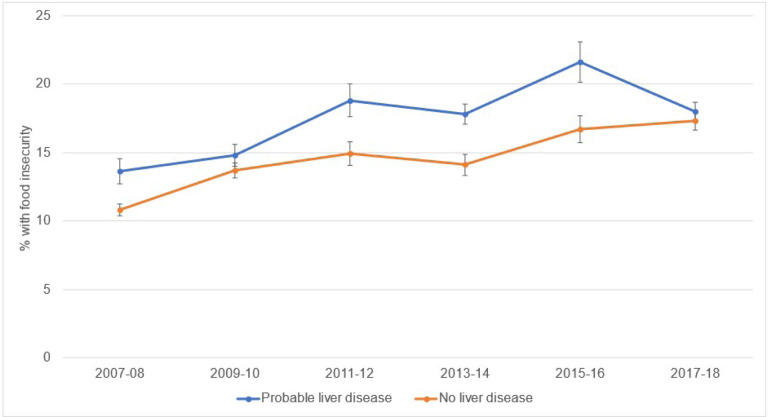
Trends in the Prevalence of Food Insecurity

Figure 1 shows trends in food insecurity among those with and without liver disease, after adjusting for sociodemographic covariates. Among adults with liver disease, the adjusted prevalence of food insecurity rose from 16.9% in 2007–2008 to 24.0% in 2017–2018 (*P-trend = 0.0003*). Among adults without liver disease, the adjusted prevalence of food insecurity increased from 13.6% in 2007–2008 to 20.4% in 2017–2018 (*P-trend *<* 0.0001*). At each survey wave, the prevalence of food insecurity was higher among those with liver disease than those without liver disease.

### Predictors of food insecurity among adults with liver disease

In Table 3, results from the multivariable model showed that the odds of food insecurity were significantly lower among those with older age (≥ 50 years), with higher educational attainment (OR 0.77, 95% CI 0.65, 0.92), and with higher incomes (OR 0.22, 95% CI 0.17, 0.29), and significantly higher among females (OR 1.26, 95% CI 1.05, 1.51), Hispanic adults (OR 1.68, 95% CI 1.30, 2.18), with BMI ≥ 30 kg/m2 (OR 1.34, 95% CI 1.02, 1.77), with elevated glycohemoglobin (OR 1.29, 95% CI 1.05, 1.58), and participating in SNAP (OR 2.53, 95% CI 2.02, 3.17).

## Discussion

Metabolic liver disease is a large and growing public health problem. Dietary modification is essential to prevent progression to cirrhosis and its complications. Common recommendations include eliminating excess carbohydrates in favor of whole foods.^6,16,17^ This strategy is challenged in the context of increasingly prevalent food insecurity. In this nationally representative study, we showed that food insecurity was prevalent among those with liver disease, and their levels increased significantly over time. Food insecurity poses a major barrier to the effectiveness of interventions to improve liver health and our data highlight both the increasing burden overall and within high-risk subgroups.

### New findings

Our study extends the literature with three key findings. First, we show that food insecurity is rising among persons with metabolic liver disease, from 16.9% in 2007–2008 and peaking at 28.7% in 2015–2016. Second, we show that among those with metabolic liver disease the subgroups with the starkest increases in food insecurity include younger-aged adults (< 50 years), women, Hispanic persons, people with less than a college education, unmarried/unpartnered persons, and those with BMI > 30 kg/m^2^. Third, we show that among adults with liver disase, food insecurity is strongly predicted by markers of economic disadvantage, including lower educational attainment, lower income, and SNAP participation, and by correlated health risks, such as elevated BMI and glycohemoglobin.

### Confirmatory findings

Our data confirms and extends prior findings showing that persons with chronic disease, such as those with cardiovascular disease, are more likely to have food insecurity.^18^ In contrast to cardiovascular disease, however, metabolic liver disease is both caused by malnutrition and, itself, independently associated with cardiovascular disease morbidity and mortalty.^19^ Fluid retention causes nausea, anorexia, and is associated with poor nutritional intake. Liver disease related cholestasis, microbiome alterations, and shunting leads to decreased absorption of macro and micro-nutrients. Patients with liver disease are managed with further dietary restrictions including salt and fluid limits leading to additional challenges obtaining nutritionally adequate and palatable diets.^20^ Comprehensive treatment of CLD must include targeted interventions for malnutrition to address both these causes and effects.

Recently, Ochoa-Allemant et al showed that among persons with metabolic liver disease, those with MetALD have a higher prevalence of food insecurity (42.1% vs. 27.7%) compared to those with MASLD.^21^ Food insecurity is also associated with increased alcohol intake,^22^ potentiating liver disease. Along with treating alcohol use disorder nutrition must also be targeted in a MetALD management strategy to decrease the rising burden of alcohol related liver disease.

### Next steps

Our data suggest that the burden of food insecurity is increasing necessitating targeted intervention. Targeted dietary therapies including those commonly prescribed for MASLD may be insufficient to address those with food insecurity. Patients with liver disease and food insecurity should be offered social work counseling and potential enrollment in federal, state and/or local programs to improve access to food. Our data demonstrates that SNAP enrollment may be insufficient and additional supplementation and counseling may be needed specifically targeting those with CLD. Those most at risk including patients < 50, women, Hispanic persons, people with less than a college education, unmarried/unpartnered persons, and those with BMI > 30 kg/m^2^ merit additional awareness for conscious targeted intervention. More studies are needed to inform what additional interventions may be most effective for these groups of patients with CLD.

### Contextual factors

These data must be considered in the context of the study design. First, advanced liver disease can only be estimated using laboratory indices which have imperfect sensitivity and specificity. Second, as a cross-sectional study, causality cannot be inferred as prior food security is unknown. However, present food security remains critical to the management of CLD. Finally, although our model adjusts for many confounders there may be unmeasured confounders such as remote alcohol intake or prior behaviors or prior health status measures such as body mass index.

## Conclusions

Interventions to address food insecurity are needed to address the growing burden of CLD. These data demonstrate that the rate of food insecurity amongst patients with CLD is rising and is associated with metabolic liver disease. Additional research is needed to stop this trend and determine how to best address nutrition in the context of CLD to promote improved food security and in turn liver health.

## Figures and Tables

**Table 1:** Demographics of Survey Waves

	Total N = 24847	2007–2008 n = 4611	2009–2010 n = 4718	2011–2012 n = 3866	2013–2014 n = 4043	2015–2016 n = 3937	2017–2018 n = 3672	*P*
	%	%	%	%	%	%	%	
Food insecure (%)	15.3	11.5	14.0	15.8	15.1	18.1	17.5	*0.0009*
Probable liver disease (%)	24.6	24	25.3	22.8	26.1	28.3	21.1	*0.85*
Advanced liver disease (%)	3.4	1.9	3.7	3.9	4.1	4.2	2.7	*0.07*
Age								*0.003*
20–29	12.6	14.2	11.9	11.2	11.0	13.3	13.9	
30–39	16.5	18.5	18.2	16.4	16.7	15.4	13.6	
40–49	20.1	21.5	21.0	20.1	20.5	19.5	17.7	
50–59	20.1	19.1	19.7	21.1	19.9	20.1	20.4	
60–69	15.7	12.8	14.7	15.8	16.8	16.5	17.6	
70–79	9.6	8.6	9.0	9.5	9.5	9.6	11.2	
≥80	5.6	5.3	5.5	5.9	5.6	5.7	5.6	
Female sex	50.3	50.1	49.5	50.2	51.0	50.1		*0.8*
Race and ethnicity (%)								*0.83*
Non-Hispanic White	67.9	71.2	70.1	68.4	67.9	65.5	64.3	
Non-Hispanic Black	10.7	10.7	10.5	11.0	10.5	10.8	11.0	
Hispanic	14.8	13.9	14.0	14.5	15.3	15.8	15.5	
Other	6.5	4.1	5.4	6.1	6.3	7.9	9.3	
High school degree or fewer years	42.1	48.5	44.9	40.6	39.6	37.7	41.5	*0.008*
Married or living with partner	64.5	65.5	65.1	62.9	64.4	65.3	63.9	*0.82*
≤ 200% federal poverty level	39.9	39.8	39.3	43.4	40.0	38.8	38.3	*0.66*
SNAP participation	15.3	10.9	13.5	17.1	16.9	16.6	16.6	*0.03*
Heavy drinker	1.1	1.0	1.3	1.8	1.6	1.1	-	
BMI ≥ 30 kg/m2	39.6	36.3	38.6	36.7	39.9	42.0	44.2	*0.0003*
Diabetes	13.8	12.2	12.5	13.1	13.7	14.7	16.5	*0.001*

**Table 2:** Prevalence of Food Insecurity By Key Subgroups

	2007–2008	2009–2010	2011–2012	2013–2014	2015–2016	2017–2018	*P-trend*
	Mean	Mean	Mean	Mean	Mean	Mean	
Age < 50 y	17.6	21.8	27.3	24.0	28.0	22.3	*0.004*
Age ≥ 50 y	9.5	8.8	11.9	12.7	16.5	14.6	*< 0.0001*
Female	14.6	16.4	18.8	19.2	22.0	21.1	*< 0.0001*
Male	12.1	12.1	18.8	15.4	21.0	14.7	*0.0007*
Non-Hispanic White	8.9	8.2	12.8	13.4	16.1	12.1	*0.0002*
Non-Hispanic Black	17.7	28.0	29.6	17.9	28.9	20.1	*0.26*
Hispanic	26.4	32.8	32.2	35.5	35.2	34.6	*0.005*
HSD or fewer yrs education	18.9	21.7	26.1	23.1	29.4	24.3	*0.0001*
Any college	8.2	8.5	12.8	13.7	16.5	13.2	*< 0.0001*
Not married or partnered	18.4	18.7	24.9	21.1	26.3	22.1	*0.0002*
Married or partnered	11.1	12.6	15.4	15.9	19.2	16.2	*0.0001*
≤ 200% FPL	25.2	29.5	33.8	33.5	39.2	38.5	*< 0.0001*
> 200% FPL	5.3	4.8	5.3	6.1	8.7	7.8	*0.003*
Diabetes	14.0	15.1	21.6	18.9	26.4	14	*0.003*
No diabetes	13.4	14.6	17.5	17.3	19.8	20	*< 0.0001*
BMI ≥ 30 kg/m2	13.7	15.3	19.9	18.6	22.5	18.1	*< 0.0001*
BMI < 30 kg/m2	13.8	10.4	11.1	12.5	13.4	16.6	*0.003*
SNAP participation	40.5	40.5	46.6	44.7	54.3	44.5	*0.09*
No SNAP participation	9.6	10.4	12.3	11.8	14	12.9	*0.0004*

**Table 3:** Association between liver disease and food insecurity

	Model 1		Model 2	
	OR	95% CI	OR	95% CI
Probable liver disease				
No	Ref.		Ref.	
Yes	**1.26**	**1.12, 1.42**	**1.21**	**1.07, 1.37**
Survey year				
2007–2008	Ref.		Ref.	
2009–2010	**1.30**	**1.02, 1.64**	1.27	0.97, 1.65
2011–2012	**1.55**	**1.18, 2.04**	**1.40**	**1.12, 1.74**
2013–2014	**1.45**	**1.12, 1.88**	**1.36**	**1.05, 1.78**
2015–2016	**1.80**	**1.37, 2.37**	**1.90**	**1.48, 2.43**
2017–2018	**1.80**	**1.43, 2.25**	**1.82**	**1.46, 2.27**
Age				
20–29	Ref.		Ref.	
30–39	0.89	0.78, 1.02	1.05	0.88, 1.25
40–49	**0.71**	**0.62, 0.83**	1.07	0.89, 1.28
50–59	**0.53**	**0.49, 0.62**	**0.81**	**0.67, 0.97**
60–69	**0.36**	**0.30, 0.43**	**0.57**	**0.47, 0.70**
70–79	**0.29**	**0.27, 0.36**	**0.40**	**0.32, 0.50**
≥80	**0.18**	**0.13, 0.25**	**0.23**	**0.17, 0.32**
Male	Ref.		Ref.	
Female	**1.18**	**1.10, 1.26**	1.06	0.97, 1.15
Non-Hispanic White			Ref.	
Non-Hispanic Black			**1.29**	**1.10, 1.50**
Hispanic			**1.74**	**1.47, 2.06**
Other			1.08	0.86, 1.35
High school degree or fewer years			Ref.	
Any college			**0.63**	**0.57, 0.70**
Married or living with partner			Ref.	
Not married or partnered			**1.33**	**1.19, 1.50**
Income to poverty ratio				
≤200% federal poverty level			Ref.	
>200% federal poverty level			**0.25**	**0.21, 0.29**
SNAP participation				
Non-participant			Ref.	
SNAP participant			**2.81**	**2.40, 3.29**

CI: confidence interval, SNAP: Supplemental Nutrition Assistance Program, OR = odds ratio

**Table 4:** Predictors of Food Insecurity Among Participants with Liver Disease

	OR	95% CI
Survey year		
2007–2008	Ref.	
2009–2010	1.13	0.75, 1.71
2011–2012	1.34	0.88, 2.05
2013–2014	1.39	0.86, 2.25
2015–2016	1.87	1.21, 2.88
2017–2018	1.71	1.03, 2.84
Age		
20–29	Ref.	
30–39	1.14	0.80, 1.61
40–49	1.11	0.83, 1.48
50–59	**0.61**	**0.45, 0.83**
60–69	**0.61**	**0.42, 0.89**
70–79	**0.36**	**0.26, 0.51**
≥80	**0.29**	**0.15, 0.55**
Male	Ref.	
Female	**1.26**	**1.05, 1.51**
Race and ethnicity		
Non-Hispanic White	Ref.	
Non-Hispanic Black	1.20	0.96, 1.51
Hispanic	**1.68**	**1.30, 2.18**
Other	1.35	0.97, 1.90
High school degree or fewer years	Ref.	
Any college	**0.77**	**0.65, 0.92**
Married or living with partner	Ref.	
Not married or partnered	1.2	0.98, 1.48
≤200% federal poverty level	Ref.	
>200% federal poverty level	**0.22**	**0.17, 0.29**
SNAP participation		
Non-participant	Ref.	
SNAP participant	**2.53**	**2.02, 3.17**
BMI < 30 kg/m2	Ref.	
≥30 kg/m2	**1.34**	**1.02, 1.77**
Diabetes/ elevated glycohemoglobin		
No	Ref.	
Yes	**1.29**	**1.05, 1.58**

CI: confidence interval, SNAP: Supplemental Nutrition Assistance Program, OR = odds ratio

## Data Availability

All data available from NHANES
